# Characteristics, management, and outcomes of segmental and subsegmental pulmonary embolism in ICU patients: A retrospective cohort study

**DOI:** 10.1371/journal.pone.0353422

**Published:** 2026-07-10

**Authors:** Nuanprae Kitisin, Nattaya Raykateeraroj, Yukiko Hikasa, Jonathan Nübel, Alessandro Caroli, Glenn Eastwood, Emily Harman, Sandra Lussier, Derick Adigbli, Jane E. Lewis, Numan Kutaiba, Daryl Jones, Sikarin Upala, Ary Serpa Neto

**Affiliations:** 1 Department of Intensive Care, Austin Hospital, Heidelberg, Victoria, Australia; 2 Department of Anesthesiology, Faculty of Medicine Siriraj Hospital, Mahidol University, Bangkok, Thailand; 3 Dipartimento di Scienze dell’Emergenza, Anestesiologiche e della Rianimazione, Fondazione Policlinico Universitario A. Gemelli IRCCS, Rome, Italy; 4 Australian and New Zealand Intensive Care Research Centre (ANZIC-RC), School of Public Health and Preventive Medicine, Monash University, Melbourne, Australia; 5 The George Institute for Global Health, University of New South Wales, Sydney, New South Wales, Australia; 6 Medical School, Faculty of Medical Sciences, University College London, London, England; 7 Department of Critical Care, School of Medicine, University of Melbourne, Parkville, Victoria, Australia; 8 Department of Radiology, Austin Hospital, Heidelberg, Victoria, Australia; 9 Department of Preventive and Social Medicine, Faculty of Medicine Siriraj Hospital, Mahidol University, Bangkok, Thailand; 10 Data Analytics Research and Evaluation (DARE) Centre, Austin Hospital, Melbourne, Australia; 11 Department of Critical Care Medicine, Hospital Israelita Albert Einstein, Sao Paulo, Brazil; King Fahd Military Medical Complex, SAUDI ARABIA

## Abstract

**Objective:**

To describe the incidence, management, and outcomes of segmental and subsegmental pulmonary embolism (PE) in intensive care unit (ICU) patients and to explore associations between therapeutic-dose anticoagulation and clinical outcomes.

**Design:**

Single-center retrospective cohort study.

**Setting:**

Tertiary academic hospital ICU between January 2019 and June 2025.

**Patients:**

Critically ill adults (≥18 years) who underwent computed tomography pulmonary angiography (CTPA) during ICU admission and had radiologically confirmed segmental or subsegmental PE.

**Interventions:**

None.

**Measurements and main results:**

Radiology reports of all CTPA examinations performed in ICU-admitted patients were screened to identify the most proximal level of thrombus. Clinical records were reviewed for demographics, illness severity, radiologic characteristics, anticoagulation practice, bleeding, venous thromboembolism (VTE) recurrence, and mortality. Among 896 CTPA examinations performed in 804 patients, 164 examinations (18.3%) identified PE. Of these, 115 scans (12.8% of all CTPAs) demonstrated distal PE only, corresponding to 104 patients (12.9%) (61 segmental, 43 subsegmental). Overall, 96% of patients with distal PE received anticoagulation and 86% of anticoagulated patients received therapeutic-dose regimens. Bleeding occurred in 15% (major bleeding 12%), 90-day VTE recurrence in 7.8%, and 90-day mortality in 24%. No statistically significant association was found between the use of therapeutic-dose anticoagulation and 90-day mortality (adjusted odds ratio [OR], 0.70; 95% CI, 0.21–2.45), bleeding episodes (adjusted OR, 2.34; 95% CI, 0.47–19.2), or VTE recurrence (adjusted OR, 0.69; 95% CI, 0.11–6.22).

**Conclusions:**

In critically ill adults, segmental and subsegmental PE are commonly detected on CTPA and are usually treated with therapeutic-dose anticoagulation. Although VTE recurrence was less frequent than bleeding episodes and mortality, our study did not find a significant association between therapeutic-dose anticoagulation and bleeding episodes, recurrent VTE, or mortality. Larger prospective studies are needed to define optimal anticoagulation strategies for ICU patients with distal PE.

## Introduction

The widespread use of multidetector computed tomography pulmonary angiography (CTPA) has markedly increased the detection of distal pulmonary emboli (PE) involving segmental and subsegmental branches [1,2]. These smaller, third- or fourth-order emboli rarely cause major hemodynamic compromise. In contrast, main or lobar PEs carry a higher thrombotic burden, greater right ventricular strain, and a higher risk of cardiovascular collapse and short-term mortality [3–5]. Consequently, while management of proximal PE is well established, the optimal approach to distal PE remains uncertain, with current guidelines reflecting this ambiguity. The 2019 European Society of Cardiology (ESC) guideline generally supports anticoagulation for confirmed PE, while acknowledging uncertainty around isolated subsegmental PE (SSPE) and emphasizing careful diagnostic confirmation [6]. Conversely, the 2021 American College of Chest Physicians (ACCP) guideline [7] recommends clinical surveillance in carefully selected low-risk patients with isolated SSPE and no proximal deep-vein thrombosis, reserving anticoagulation for those at higher recurrence risk.

Current available evidence specific to distal PE remains sparse. A single-center retrospective study from our institution examining all patients identified 166 patients with SSPE (3% of all CTPAs) over three years (2017–2019), with higher long-term mortality in SSPE observed regardless of anticoagulation strategy [8]. A retrospective study of 43 patients found that more than half of patients with isolated SSPE received anticoagulation, no recurrences occurred, but two experienced major bleeding events [9]. Similarly, another study reported very low recurrence (3.1%) and bleeding (0.7%) rates in untreated SSPE [10], and a systematic review highlighted the absence of randomized trials comparing anticoagulation with surveillance, concluding that the net benefit of treatment remains uncertain [11]. Moreover, segmental and subsegmental PE may differ in clot burden, hemodynamic impact, and uncertainty regarding the benefit of anticoagulation, particularly in critically ill patients where bleeding risk is high [12]. Registry studies also reported lower mortality and fewer adverse events in segmental and SSPE compared with proximal PE, but these cohorts largely excluded critically ill patients [4,13]. In the intensive care unit (ICU), this uncertainty is amplified as patients have a markedly higher thrombotic risk from immobility, inflammation, and invasive procedures [14–17], but also a substantial bleeding risk due to organ dysfunction and anticoagulant exposure [16,18,19]. Moreover, tachypnea, hypoxemia, and tachycardia, common PE symptoms, are ubiquitous yet nonspecific in critical illness [14,16], leading to a more liberal CTPA use and potentially different PE phenotypes. A small observational study suggested increased short-term mortality in ICU patients with isolated SSPE [20]. This potentially heightened mortality risk, coupled with the lack of available evidence in the management of this patient population, may at least partly explain why anticoagulation is initiated and prolonged despite competing bleeding risks.

To date, no study has specifically described the management or outcomes of segmental or SSPE in ICU patients. Given the absence of ICU specific evidence and the high risks of both undertreatment and overtreatment of distal PE in this population, defining how these events are currently managed is an essential first step toward generating evidence-based treatment strategies. This observational study aimed to characterize the clinical features, anticoagulation practices, clinical outcomes, and to explore associations between therapeutic-dose anticoagulation and clinical outcomes among ICU patients diagnosed with segmental or SSPE on CTPA ordered during ICU stay.

## Methods

### Study design and ethical approval

This single-center, retrospective cohort study was conducted at Austin Hospital, a tertiary academic center in Melbourne, Australia. The study was conducted in a single mixed medical–surgical intensive care unit. Ethics approval was granted by the Austin Health Human Research Ethics Committee (HREC/25188/Austin-2025), in accordance with the National Statement on Ethical Conduct in Human Research (2023) (approval date 11 August 2025). The requirement for individual informed consent was waived due to the retrospective design and use of routinely collected clinical data. Investigators had access to identifiable information during data extraction; however, all data were de-identified prior to analysis.

### Study population

The source population comprised all critically ill adult patients (≥18 years) who underwent at least one CTPA during an ICU admission between 1 January 2019 and 30 June 2025. Using the institutional radiology information system, we retrieved all CTPA examinations for patients admitted to the ICU during the study period and cross-referenced these with ICU admission records to identify scans performed between ICU admission and ICU discharge. All corresponding radiology reports were then manually screened by the investigators.

Patients were included if at least one CTPA obtained during ICU stay demonstrated a radiologically confirmed segmental or subsegmental pulmonary embolism (SSPE). In patients with multiple CTPAs during the same ICU admission, the first scan with a positive PE report was designated as the index CTPA, and the final radiologist interpretation of that study was used to confirm PE and determine the most proximal anatomical level of thrombus. Segmental PE was defined as thrombus involving a segmental pulmonary arterial branch, with or without additional subsegmental extension, whereas SSPE was defined as thrombus confined to subsegmental branches, without involvement of more proximal vessels [21,22]. Segmental and subsegmental PE were analyzed together as ‘distal PE,’ defined as thrombus distal to the lobar pulmonary arteries, while also being reported as separate subgroups throughout. Although current guidelines, including the ACCP CHEST guideline [7], address subsegmental but notsegmental PE explicitly, both entities lie distal to the lobar vessels, and ICU-specific evidence regarding anticoagulation strategies for these smaller emboli remains limited. To preserve transparency, all analyses were additionally stratified by anatomical level (segmental vs. subsegmental). Patients with known chronic thromboembolic pulmonary disease and those with main-trunk or lobar pulmonary emboli were excluded.

### Data collection

Data were extracted from the electronic medical record, radiology information system, pathology databases, and pharmacy dispensing records using a standardized case report form to ensure consistency and completeness. Collected variables included demographic characteristics, comorbidities, the worst physiologic values within six hours of the index CTPA, and laboratory markers obtained within 24 hours after the index CTPA. For laboratory markers with multiple measurements during this period, the closest available value after CTPA was used to describe the early biochemical profile following PE diagnosis. Formal pre-test probability scores, such as the Wells or Geneva scores, were not routinely documented in the medical record and therefore could not be reliably reconstructed. Instead, the documented clinical features or indications prompting CTPA were extracted from clinical notes and radiology requests, including hypoxia, tachycardia, tachypnea, dyspnoea, chest pain, hypotension, elevated D-dimer, hemoptysis, or other documented concerns.

Radiological data included the anatomical level of PE (segmental or subsegmental), the distribution of emboli (single-lobe, multiple unilateral, or bilateral involvement), the presence of right ventricular dilation or strain (identified on CTPA or echocardiography), and concurrent lower-limb deep-vein thrombosis (DVT) on ultrasound. Ancillary CTPA findings such as atelectasis, consolidation, pulmonary edema, and other pulmonary infiltrates were also captured.

Management-related variables encompassed anticoagulation practices both during ICU admission and following discharge. For all patients, anticoagulation categories were defined as follows: 1) prophylactic anticoagulation prior to CTPA, defined as prophylactic anticoagulation administered according to the institutional venous thromboembolism (VTE) prophylaxis protocol; 2) therapeutic anticoagulation prior to CTPA, defined as therapeutic anticoagulation given for another indication, such as prior VTE or atrial fibrillation (patients already receiving therapeutic anticoagulation prior to CTPA were classified as receiving therapeutic anticoagulation for PE only if the same regimen was continued unchanged after PE confirmation); 3) any anticoagulation for PE, defined as prophylactic or therapeutic anticoagulation initiated or continued following PE confirmation on CTPA; and 4) therapeutic-dose anticoagulation for PE, defined as therapeutic anticoagulation (such as enoxaparin 1 mg/kg twice daily or unfractionated heparin infusion titrated to target activated partial thromboplastin time [aPTT]) initiated or continued following PE confirmation on CTPA.

Additional data included the anticoagulant agent class, dosing regimen, route of administration, timing of initiation, and whether therapy was continued after hospital discharge. Use of inferior vena cava (IVC) filters and thrombolytic interventions, either systemic or catheter-directed, was also recorded.

### Outcomes

The primary outcome was the frequency of segmental and SSPE among ICU patients who underwent CTPA for any indication during the study period. Secondary outcomes included: 1) management practices, including the proportion of patients receiving anticoagulation, time to initiation of anticoagulation, dosing protocol, and agent selected during ICU stay and at hospital discharge; and 2) clinical outcomes, including mortality, recurrent VTE, and bleeding complications within 90 days from the index scan.

Recurrent VTE was defined as new, objectively confirmed PE or DVT within 90 days from the index scan. Bleeding events were identified from clinical, procedural, and laboratory records, and classified by Bleeding Academic Research Consortium (BARC) criteria (detailed in S1 File), with major bleeding classified when BARC ≥ 3 [23]. Mortality outcomes included in-hospital, 30-day, and 90-day all-cause mortality, and additional clinical outcomes included ICU and hospital length of stay, and ICU readmission during the same hospitalization.

### Statistical analysis

Categorical variables were summarized as counts and percentages and compared using the χ² test or Fisher’s exact test, as appropriate. Continuous variables were reported as medians with interquartile ranges (IQRs) and compared using the Wilcoxon rank-sum test. Missing data were reported as “unspecified.” Analyses were performed on available cases without imputation.

For major clinical outcomes related to therapeutic anticoagulation, prespecified multivariable logistic regression models were used to estimate adjusted odds ratios (aORs) with 95% confidence intervals (CIs), adjusting for clinically relevant covariates selected a priori, including age, active cancer, chronic kidney disease, right ventricular dilation, and concomitant DVT. Adjusted treatment-association analyses were performed in the overall cohort and, where estimable, separately within segmental and SSPE subgroups. Absolute risks and absolute risk differences were also calculated using the Newcombe hybrid score method with Wilson intervals, both in the full cohort and within each PE subgroup.

Associations between clinical variables and key secondary outcomes, including any bleeding and recurrent VTE, were first examined using univariable logistic regression, with results expressed as odds ratios (ORs) and 95% CIs. Because only eight recurrence events occurred, no multivariable predictor model for 90-day VTE recurrence was performed beyond the prespecified treatment-association model. For bleeding outcomes, variables with a univariable p < 0.10 were entered into Firth penalized logistic regression models to obtain bias-reduced estimates in the presence of sparse events (16 bleeding events). Subgroup analyses by admission type were not performed because of the limited number of patients and outcome events within each subgroup.

The study enrolment period was defined a priori, and all consecutive eligible patients during this interval were included. Because the primary aim was descriptive, sample size adequacy was assessed using a screening-based approach rather than a hypothesis-testing framework, consistent with prior subsegmental PE cohort studies [9]. Based on prior institutional data showing that isolated subsegmental PE accounted for approximately 3% of all hospital-wide CTPA examinations [8], and considering that the present study included both segmental and subsegmental PE in an ICU population, we anticipated that approximately 10–12% of ICU CTPA examinations would demonstrate distal PE. Therefore, screening approximately 850–1,000 CTPA examinations was expected to identify approximately 100 patients with segmental or subsegmental PE, a sample considered sufficient to describe frequency, management, and clinical outcomes. No separate sample size calculation was performed for exploratory comparisons of anticoagulation intensity and clinical outcomes; these analyses were limited by the number of outcome events and should be interpreted as hypothesis-generating. All statistical analyses were performed using R version 4.4.3. For patient-level analyses, the unit of analysis was the individual patient. Statistical significance was defined as a two-sided p < 0.05.

## Results

### Patient characteristics

Between 01 January 2019 and 30 June 2025, 896 CTPA scans were performed in 804 patients during ICU admission (S1 Fig in S1 File). Of these, 732 (81.7%) showed no PE. Among the 164 scans with confirmed PE (18.3%), 49 involved main-trunk or lobar emboli and were excluded. The remaining 115 scans (12.8%) demonstrated distal PE; 68 segmental (7.6%) and 47 SSPE (5.2%). After accounting for multiple scans per patient, the final cohort comprised 104 unique patients: 61 with segmental PE and 43 with SSPE.

Baseline characteristics are shown in Table 1. The median (IQR) age was 61.5 years (50.5–69.5), and 37% of patients were female. The cohort represented a mixed ICU population, comprising 66 medical admissions, 22 elective surgical admissions, and 16 emergency surgical admissions. Chronic illness was common, most frequently hypertension (38%) and diabetes (21%). Median APACHE II and III scores were 17 and 57, respectively, with no differences between segmental and subsegmental groups. Physiological variables within 6 hours of CTPA, including heart rate, mean arterial pressure, respiratory rate, and PaO_2_/FiO_2_ ratio, were also similar between groups. Presenting features were heterogeneous and non-specific, most often hypoxia (33%), tachycardia (31%), and tachypnea (15%). Laboratory markers, including lactate, creatinine, D-dimer, troponin, and NT-proBNP were comparable across groups.

**Table 1:** Baseline Characteristics of ICU Patients with Subsegmental and Segmental Pulmonary Embolism.

	Total cohort (N = 104)	SSPE (n = 43)	Segmental PE (n = 61)	p-value
Age, years	61.5 [50.5, 69.5]	64.0 [51.0, 71.0]	59.0 [49.0, 68.0]	0.077
Sex — n (%)				0.68
Female	38 (37%)	17 (40%)	21 (34%)	
Male	66 (63%)	26 (60%)	40 (66%)	
BMI, kg/m²	28.8 [24.0, 34.2]	28.7 [23.8, 33.3]	29.2 [24.2, 34.7]	0.63
Comorbidities — n (%)				
Hypertension	40 (38%)	17 (40%)	23 (38%)	0.99
Diabetes mellitus	22 (21%)	5 (12%)	17 (28%)	0.054
Coronary artery disease	13 (13%)	5 (12%)	8 (13%)	0.99
Active cancer	15 (14%)	7 (16%)	8 (13%)	0.78
Chronic kidney disease	4 (4%)	2 (5%)	2 (3%)	0.99
Atrial fibrillation	8 (8%)	3 (7%)	5 (8%)	0.99
Immunocompromised state	4 (4%)	1 (2%)	3 (5%)	0.64
Chronic lung disease	14 (13%)	7 (16%)	7 (11%)	0.56
Currently on oral anticoagulant	8 (8%)	2 (5%)	6 (10%)	0.46
Severity score				
APACHE II score	17 [13, 22]	17 [13, 24]	16 [13, 21]	0.36
APACHE III score	57 [43, 72]	61 [44, 74]	53 [40, 71]	0.45
Type of admission – n (%)				
Surgical Elective	22 (21%)	9 (21%)	13 (21%)	0.99
Surgical Emergency	16 (15%)	5 (12%)	11 (18%)	0.42
Medical	66 (63%)	29 (67%)	37 (61%)	0.54
Physiology within 6h of CTPA				
Highest heart rate, bpm	110.5 [95.5, 125.0]	110.0 [95.0, 125.0]	111.0 [100.0, 125.0]	0.34
Lowest mean arterial pressure, mmHg	70.0 [65.5, 77.5]	70.0 [64.0, 76.0]	70.0 [66.0, 78.0]	0.60
Highest respiratory rate, /min	25.0 [20.0, 31.0]	26.0 [21.0, 29.0]	25.0 [20.0, 32.0]	0.73
Lowest PaO₂/FiO₂ ratio	188.0 [134.0, 271.4]	185.0 [160.0, 244.0]	192.6 [130.0, 297.4]	0.61
Primary documented indication for CTPA— n (%)				0.027
Asymptomatic	5 (5%)	1 (2%)	4 (7%)	
Chest pain	5 (5%)	4 (9%)	1 (2%)	
Dyspnea	3 (3%)	2 (5%)	1 (2%)	
Hemoptysis	1 (1%)	1 (2%)	0 (0%)	
High D-dimer	3 (3%)	3 (7%)	0 (0%)	
Hypotension	3 (3%)	1 (2%)	2 (3%)	
Hypoxia	34 (33%)	14 (33%)	20 (33%)	
Tachycardia	32 (31%)	14 (33%)	18 (30%)	
Tachypnea	16 (15%)	2 (5%)	14 (23%)	
Other	2 (2%)	1 (2%)	1 (2%)	
Laboratory markers within 24 h after CTPA				
Lactate, mmol/L	2.10 [1.40, 2.80]	1.70 [1.30, 2.80]	2.20 [1.55, 3.20]	0.10
Serum creatinine, μmol/L	84.0 [67.0, 119.0]	83.0 [67.0, 118.0]	86.00 [67.0, 128.0]	0.93
D-dimer collected – n (%)	18 (17%)	10 (23%)	8 (13%)	0.20
D-dimer, µg/L	4,272.50 [2,053.00, 15,082.00]	2,822.50 [1,496.00, 10,333.00]	8,969.00 [4,272.50, 15,846.50]	0.083
Troponin collected – n (%)	49 (47%)	20 (47%)	29 (48%)	0.99
Troponin, ng/L	16.00 [5.00, 57.00]	9.50 [4.50, 66.00]	19.00 [7.00, 57.00]	0.58
NT proBNP collected – n (%)	14 (13%)	3 (7%)	11 (18%)	0.15
NT-proBNP, pg/mL	1,197.50 [70.00, 2,829.00]	607.00 [67.00, 1,204.33]	1,788.00 [70.00, 2,829.00]	0.88

Data are presented as median [IQR] or n (%). P values were calculated using the Wilcoxon rank-sum test for continuous variables and the χ² test or Fisher’s exact test for categorical variables, as appropriate. Abbreviations: APACHE = Acute Physiology and Chronic Health Evaluation; BMI = body mass index; bpm = beats per minute; CTPA = computed tomography pulmonary angiography; FiO₂ = fraction of inspired oxygen; IQR = interquartile range; MAP = mean arterial pressure; NT-proBNP = N-terminal pro-B-type natriuretic peptide; PaO₂ = partial pressure of arterial oxygen; PE = pulmonary embolism; SSPE = subsegmental pulmonary embolism.

### Radiologic findings and provoking factors

Radiological findings and provoking factors are summarized in Table 2. Most emboli were confined to a single lobe (60%), with 14% showing multiple unilateral and 26% bilateral involvement. Right ventricular dilatation on CTPA was more common in segmental than SSPE (16% vs 2%; p = 0.027). Concomitant DVT occurred in 20% of patients, with similar rates across groups. Other chest CT findings were frequent, including consolidation (29%), atelectasis (11%), and pleural effusion (10%), without differences between groups. Recent surgery (34%), immobility (33%), and COVID-19 infection (17%) were the most common potential provoking factors. At the time of CTPA, 46% of patients were mechanically ventilated and 35% were receiving vasopressor support.

**Table 2:** Radiologic Findings, Provoking Factors, and Organ-Support Characteristics at the Time of CTPA.

	Total cohort (N = 104)	SSPE (n = 43)	Segmental PE (n = 61)	p-value
PE distribution pattern – n (%)^†^				0.059
Single lobe	61 (60%)	30 (71%)	31 (52%)	
Multiple unilateral	14 (14%)	6 (14%)	8 (13%)	
Multiple bilateral	27 (26%)	6 (14%)	21 (35%)	
Right ventricular dilation – n (%)				
On CTPA	11 (11%)	1 (2%)	10 (16%)	0.027
On echocardiography	10 (10%)	2 (5%)	8 (13%)	0.19
Concomitant DVT – n (%)	21 (20%)	8 (19%)	13 (21%)	0.81
Other chest CT findings – n (%)				0.58
Consolidation	30 (29%)	14 (33%)	16 (26%)	
Atelectasis	11 (11%)	6 (14%)	5 (8.2%)	
Effusion	10 (10%)	3 (7%)	7 (11%)	
Other	53 (51%)	20 (47%)	33 (54%)	
Possible provoke factor				
Recent surgery	35 (34%)	15 (35%)	20 (33%)	0.84
COVID-19 diagnosis	18 (17%)	8 (19%)	10 (16%)	0.80
Immobility	34 (33%)	13 (30%)	21 (34%)	0.68
Cancer	7 (7%)	3 (7%)	4 (7%)	0.99
Other	1 (1.0%)	0 (0%)	1 (2%)	0.99
Organ Support at time of CTPA				
Mechanical ventilation – n (%)	48 (46%)	18 (42%)	30 (49%)	0.55
Ventilation days	8.0 [3.5, 16.0]	6.0 [2.0, 12.0]	12.0 [4.0, 19.0]	0.13
Advanced non-invasive respiratory support^‡^ – n (%)	28 (27%)	11 (26%)	17 (28%)	0.82
Low flow oxygen – n (%)	8 (8%)	5 (12%)	3 (5%)	0.27
Room air – n (%)	20 (19%)	9 (21%)	11 (18%)	0.80
Vasopressors use – n (%)	36 (35%)	14 (33%)	22 (36%)	0.83

Data are presented as median [IQR] or n (%).^†^ n=102 (2 patients with missing PE lobar distribution data); SSPE n=42, Segmental n=60. ^‡^Includes high-flow nasal cannula and non-invasive ventilation. P values were calculated using the Wilcoxon rank-sum test for continuous variables and the χ² test or Fisher’s exact test for categorical variables, as appropriate. Abbreviations: CT = computed tomography; CTPA = computed tomography pulmonary angiography; DVT = deep-vein thrombosis; HFNC = high-flow nasal cannula; IQR = interquartile range; PE = pulmonary embolism; RV = right ventricle/right-ventricular; SSPE = subsegmental pulmonary embolism.

### Anticoagulation management

**Fig 1:**
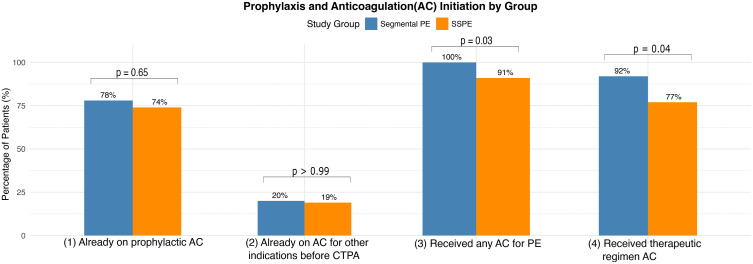
Prophylaxis and Anticoagulation Initiation by Pulmonary Embolism Group.

Anticoagulation practices varied between groups (Table 3 and Fig 1). Before CTPA, 77% of patients had received prophylactic anticoagulation and 19% were already treated with therapeutic doses for other indications, with similar rates across groups. Among the 20 patients receiving therapeutic-dose anticoagulation before CTPA, indications included COVID-19–associated coagulopathy managed with therapeutic- or intermediate-dose enoxaparin per institutional protocol (n = 5), other established venous thrombosis (n = 5), atrial fibrillation (n = 2), prior or unprovoked VTE on long-term anticoagulation (n = 1), and patient-specific or empirical indications (n = 7). After diagnosis, all segmental PE patients received anticoagulation compared with 91% of SSPE patients (p = 0.027). Among patients who received anticoagulation for PE, 86% received therapeutic-dose regimens, more commonly in segmental PE than in SSPE (92% vs 77%; p = 0.044). Low molecular weight heparin was the predominant initial agent (74%), followed by unfractionated heparin infusion (24%), with SSPE patients more often receiving LMWH (87% vs 66%; p = 0.051) (S2 Fig in S1 File). Therapeutic targets were achieved in 63% of patients, more often in the segmental group (75% vs 44%; p = 0.003). Episodes of supra-therapeutic levels of anticoagulation occurred in 25% of patients, without differences between groups.

**Table 3:** Anticoagulation Management and Other Interventions in Patients with Subsegmental Versus Segmental Pulmonary Embolism.

	Total cohort (N = 104)	SSPE (n = 43)	Segmental PE (n = 61)	p-value
Received prophylactic anticoagulation – n (%)	79 (77%)	32 (74%)	47 (78%)	0.65
On therapeutic anticoagulation for another indication – n (%)	20 (19%)	8 (19%)	12 (20%)	0.99
Anticoagulation initiation – n (%)				
Any anticoagulation administered for PE	100 (96%)	39 (91%)	61 (100%)	0.027
Therapeutic regimen	86 (86%)	30 (77%)	56 (92%)	0.044
Anticoagulant type (first regimen) – n (%)				0.051
Low molecular weight heparin	74 (74%)	34 (87%)	40 (66%)	
Unfractionated heparin infusion	24 (24%)	5 (13%)	19 (31%)	
Direct oral anticoagulation	1 (1%)	0 (0%)	1 (2%)	
Other	1 (1%)	0 (0%)	1 (2%)	
Anticoagulation level achieved – n (%)	63 (63%)	17 (44%)	46 (75%)	0.003
Supra-therapeutic levels of anticoagulation	18 (25%)	5 (23%)	13 (26%)	0.99
Regimen at hospital discharge				
Continued anticoagulation after discharge – n (%)	72 (71%)	30 (73%)	42 (70%)	0.82
Regimen type (among continuers) – n (%)				0.55
Unfractionated heparin	1 (1%)	0 (0%)	1 (2%)	
Low molecular weight heparin	10 (14%)	4 (13%)	6 (15%)	
Apixaban	51 (71%)	24 (80%)	27 (64%)	
Rivaroxaban	6 (9%)	1 (3%)	5 (12%)	
Warfarin	4 (6%)	1 (3%)	3 (7%)	
Planned treatment duration (among continuers) – n (%)				0.43
≤ 1 month	3 (4%)	3 (10%)	0 (0%)	
3 months	20 (28%)	7 (23%)	13 (31%)	
4-6 months	5 (7%)	3 (10%)	2 (5%)	
6 months	11 (15%)	3 (10%)	8 (19%)	
6 months–1 year	1 (1%)	0 (0%)	1 (2%)	
1 year	5 (7%)	3 (10%)	2 (5%)	
1-2 years	1 (1%)	1 (3%)	0 (0%)	
> 2 years	3 (4%)	2 (7%)	1 (2%)	
Lifelong	20 (28%)	7 (23%)	13 (31%)	
Not specified	3 (4%)	1 (3%)	2 (5%)	
Other interventions – n (%)				
IVC filter inserted	4 (4%)	1 (2%)	3 (5%)	0.64
Concomitant antiplatelet therapy	11 (11%)	5 (12%)	6 (10%)	0.76

Data are presented as n (%) unless otherwise indicated. Percentages were calculated using non-missing denominators for each variable. Anticoagulant type, therapeutic regimen, and level achievement were calculated among patients with an initial anticoagulation regimen. Supra-therapeutic anticoagulation levels were calculated among patients with available anticoagulation-level monitoring. Continued anticoagulation after discharge was calculated among patients with available discharge-anticoagulation data; among hospital survivors, anticoagulation was continued in 72 of 83 patients (87%). Discharge regimen type and planned treatment duration were calculated among patients continuing anticoagulation at hospital discharge. P values were calculated using Fisher’s exact test for categorical variables. Level achieved was defined as at least one activated partial thromboplastin time (aPTT) or anti-Xa measurement within the institutional therapeutic range after anticoagulation initiation. Over-level was defined as any aPTT or anti-Xa value exceeding the therapeutic range during therapy. Abbreviations: CTPA = computed tomography pulmonary angiography; DOAC = direct oral anticoagulant; IQR = interquartile range; IVC = inferior vena cava; LMWH = low-molecular-weight heparin; PE = pulmonary embolism; SSPE = subsegmental pulmonary embolism; UFH = unfractionated heparin.

Comparison of anticoagulation use between patients with segmental pulmonary embolism (PE) and subsegmental PE (SSPE). Bars show the proportion of patients who (1) were already on prophylactic AC, (2) were already on AC for other indications before CTPA, (3) received any AC for PE, and (4) received a therapeutic AC regimen. P values shown above the bars indicate between-group comparisons based on chi-square tests. Abbreviation: AC = anticoagulation; PE = pulmonary embolism; SSPE = subsegmental pulmonary embolism; CTPA = computed tomography pulmonary angiography.

At discharge, anticoagulation was continued in 72 of 101 patients with available discharge-anticoagulation data (71%). Among the 83 patients who survived to hospital discharge, anticoagulation was continued in 72 patients (87%). Among those continuing therapy, apixaban was most frequently prescribed (71%), followed by low molecular weight heparin (14%) and rivaroxaban (9%). Planned treatment duration varied widely: 28% for 3 months, 15% for 6 months, and 28% lifelong (S2 Fig in S1 File). IVC filters were inserted in 4% of patients, and 11% received concomitant antiplatelet therapy.

### Clinical outcomes

Clinical outcomes are summarized in Table 4. Bleeding occurred in 15% of patients, including major bleeding in 12% (S1 Table in S1 File). Rates were similar between segmental and SSPE (16% vs 14%; p = 0.79). Bleeding sites most commonly included hemoptysis (25%), intraabdominal (19%), retroperitoneal (13%), external bleeding (13%), and intramuscular (13%). Among patients with bleeding, 69% required red blood cell transfusion and 44% required an intervention.

**Table 4:** Clinical Outcomes, Complications, and Resource Use in Patients with Subsegmental Versus Segmental Pulmonary Embolism.

	Total cohort (N = 104)	SSPE (n = 43)	Segmental PE (n = 61)	p-value
Bleeding – n (%)				
Any bleeding after CTPA	16 (15%)	6 (14%)	10 (16%)	0.79
Major bleeding (BARC ≥3)	12 (12%)	3 (7%)	9 (15%)	0.35
Site of bleeding				0.66
Gastrointestinal	1 (6%)	0 (0%)	1 (10%)	
Retroperitoneal	2 (13%)	2 (33%)	0 (0%)	
Intracerebral	1 (6%)	0 (0%)	1 (10%)	
Intraabdominal	3 (19%)	1 (17%)	2 (20%)	
Intrathoracic	1 (6%)	0 (0%)	1 (10%)	
Hemoptysis	4 (25%)	2 (33%)	2 (20%)	
External	2 (13%)	1 (17%)	1 (10%)	
Intramuscular	2 (13%)	0 (0%)	2 (20%)	
RBC transfusion required	11 (69%)	3 (50%)	8 (80%)	0.60
Intervention required	7 (44%)	2 (33%)	5 (50%)	0.63
Recurrent VTE – n (%)				
During same hospital admission	6 (6%)	1 (2%)	5 (8%)	0.40
Within 90 days	8 (8%)	2 (5%)	6 (10%)	0.47
Mortality and length of stay				
In-hospital mortality – n (%)	21 (20%)	7 (16%)	14 (23%)	0.46
90-day mortality – n (%)	24 (24%)	7 (17%)	17 (30%)	0.16
ICU length of stay, days	7.2 [3.3, 16.3]	6.2 [3.3, 10.4]	9.1 [3.4, 20.7]	0.11
Hospital length of stay, days	21.8 [12.3, 41.5]	17.4 [9.9, 37.1]	26.4 [14.1, 43.5]	0.26

Data are presented as median [IQR] or n (%). Percentages were calculated using non-missing denominators for each variable. Recurrent VTE within 90 days was calculated using the 90-day recurrence analysis set (overall, 8/102 [8%]; SSPE, 2/42 [5%]; segmental PE, 6/60 [10.0%]). Bleeding-site percentages were calculated among patients with classifiable bleeding-site data, whereas red blood cell transfusion and intervention requirement were calculated among patients with documented bleeding. P values were calculated using the Wilcoxon rank-sum test for continuous variables and Fisher’s exact test for categorical variables. Abbreviations: BARC = Bleeding Academic Research Consortium; CTPA = computed tomography pulmonary angiography; GI = gastrointestinal; ICU = intensive care unit; IQR = interquartile range; PE = pulmonary embolism; RBC = red blood cell; SSPE = subsegmental pulmonary embolism; VTE = venous thromboembolism.

Recurrent VTE was diagnosed in 6% of patients during the index admission and 8% within 90 days, with no difference between segmental and SSPE (10% vs 5%; p = 0.47) (S2 Table in S1 File). In-hospital mortality was 20% and 90-day mortality 24%, with six patients having unknown 90-day status. 90-day mortality was similar between segmental and SSPE (30% vs 17%; p = 0.16). Median ICU and hospital lengths of stay were 7.2 days (3.3–16.3) and 21.8 days (12.3–41.5), respectively, with no significant differences between groups.

### Secondary analyses

**Fig 2:**
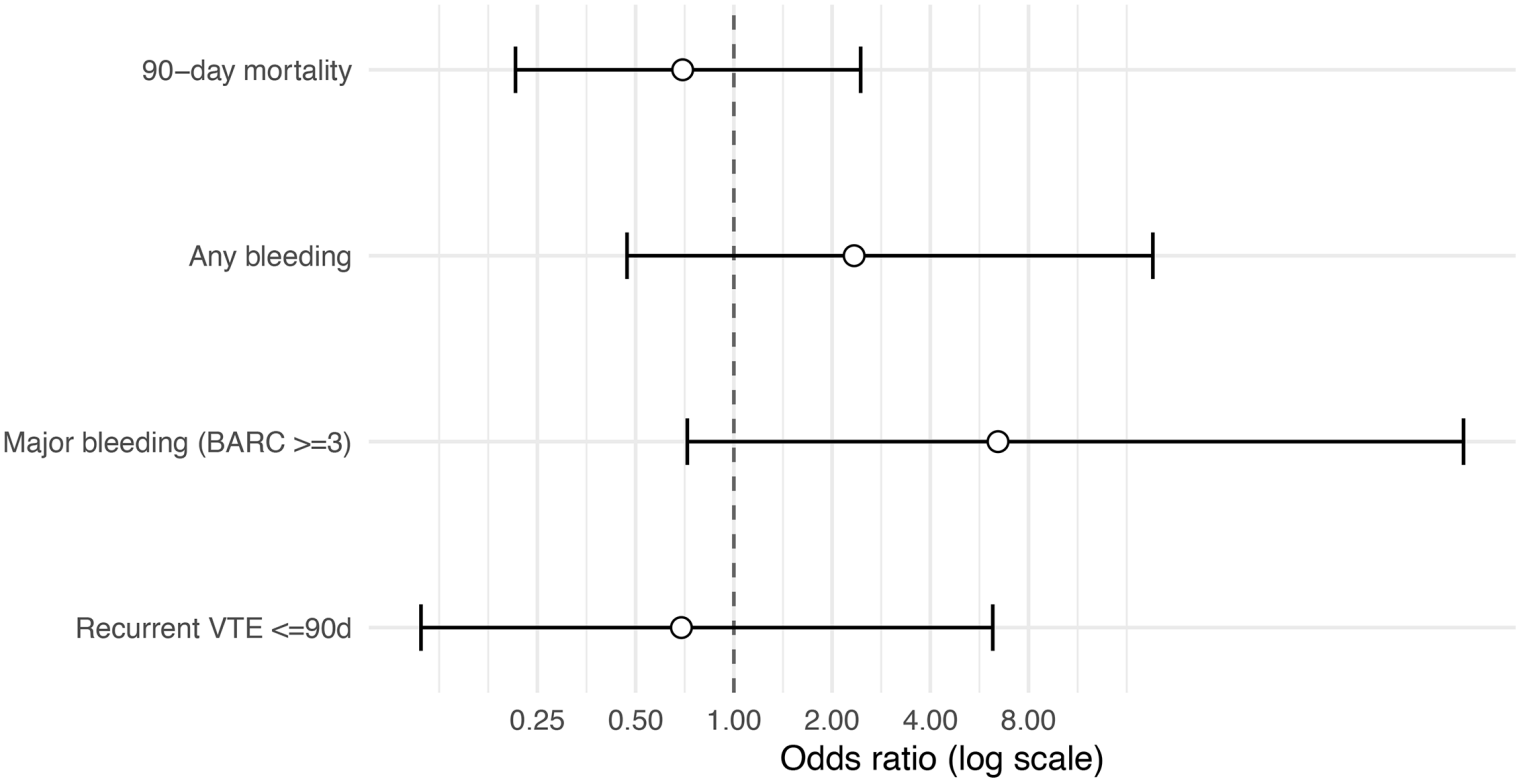
Adjusted Odds Ratios for Major Clinical Outcomes Associated with Therapeutic Anticoagulation.

Multivariable analyses showed no significant association between therapeutic anticoagulation and 90-day mortality, recurrent VTE, or bleeding (Fig 2, S3 Table in S1 File). Absolute risk differences similarly showed no significant association in the overall cohort or within segmental and SSPE subgroups (S3 and S4 Figs, S4 and S5 Tables in S1 File). In univariable analyses, right ventricular dilatation was associated with higher 90-day VTE recurrence, while no association was detected for anticoagulation intensity; however, this exploratory finding was based on few recurrence events (S6 Table in S1 File). For bleeding outcomes, right ventricular dilatation and anticoagulant class were associated with higher risk of bleeding in univariable models, though these associations attenuated in penalized regression (S7–S8 Tables in S1 File). Sensitivity analyses comparing therapeutic versus non-therapeutic dosing showed no significant differences in bleeding, VTE recurrence, or ICU length of stay; the higher mortality observed among non-therapeutically treated SSPE patients (S9 Table in S1 File) likely reflects confounding and small sample size.

Forest plot of adjusted effect estimates with 95% confidence intervals comparing therapeutic with non-therapeutic anticoagulation. Models were adjusted for age, active cancer, chronic kidney disease, right-ventricular dilatation, and concomitant deep-vein thrombosis. Estimates are displayed on a logarithmic scale; the vertical line denotes the null value. Values <1 favor therapeutic anticoagulation. Event counts (analysis set used in the models): 90-day mortality 24/98; Any bleeding 16/104; Major bleeding (BARC ≥3) 12/104; Recurrent VTE ≤ 90 days 8/102. Outcome ascertainment at 90 days: Mortality — 24/104 died, 74/104 alive, 6/104 unknown. Recurrence — 8/102 recurred, 72/102 no recurrence, 2/102 no record; 20/102 died before day 90 (competing event). Abbreviations: BARC, Bleeding Academic Research Consortium; CKD, chronic kidney disease; DVT, deep-vein thrombosis; OR, odds ratio; RV, right ventricle; VTE, venous thromboembolism.

## Discussion

### Summary of key findings

In this retrospective cohort of critically ill adults undergoing CTPA, segmental and SSPE were diagnosed in more than one in ten investigations. Anticoagulation was used in almost all patients, and most were treated using therapeutic dosing regimens regardless of embolus location. Bleeding and VTE recurrence events were infrequent, however, bleeding occurred nearly twice as often as recurrence. Across adjusted analyses, absolute risk differences, and subgroup evaluations, there was no statistically significant association between therapeutic dose anticoagulation and lower mortality, reduced recurrence, or increased bleeding, and right ventricular dilatation was the only factor linked to recurrent VTE in exploratory univariable analysis. These estimates should be interpreted with caution because the analysis was underpowered to rule out clinically important effects given the small number of outcome events and the resulting wide confidence intervals, which limit the precision of the models. Accordingly, these findings should be interpreted as descriptive and hypothesis-generating rather than definitive evidence regarding the optimal anticoagulation strategy for distal PE in ICU patients.

### Comparing with previous studies and interpretation

To our knowledge, this is the first study to specifically describe segmental and SSPE in a large ICU population. This study included consecutive adult patients admitted to the ICU who underwent CTPA for suspected acute pulmonary embolism during hospitalization. Indications for ICU admission were related to primary medical or surgical illnesses rather than PE itself, and PE was frequently detected during evaluation for hypoxemia, unexplained respiratory deterioration, or hemodynamic instability. The frequency of SSPE in our cohort was slightly higher than, but broadly consistent with, rates reported in non-ICU settings, where SSPE typically accounts for approximately 3–5% of CTPA examinations [24]. In contrast, the overall proportion of distal PE (segmental + SSPE) in our ICU cohort was comparatively high. This likely reflects the lower threshold for CTPA in critical illness, where hypoxemia, tachycardia, and tachypnea frequently prompt imaging, as well as the prothrombotic environment of the ICU, including inflammation, endothelial dysfunction, immobility, and frequent invasive procedures [14–17].

The absence of major clinical differences between segmental and subsegmental PE in this cohort likely reflects the dominant influence of underlying critical illness rather than embolic burden. The 90-day VTE recurrence rate in our cohort was 8%, with a numerically higher rate in segmental PE (10%) than in SSPE (5%). These values are slightly higher than those reported in non-ICU SSPE populations, where recurrence is generally reported at or below 3–4% [8–10,24]. In our analysis, right ventricular dilatation on CTPA was the only factor associated with recurrence; neither multilobar SSPE nor age showed an association, unlike in previous studies [10]. Markers of PE severity in our study were infrequently present. This finding suggests that mortality was primarily driven by underlying critical illness and comorbidities rather than PE severity and underlying cardiopulmonary reserve may be more relevant to recurrence risk than clot location alone [25]. Interpretation is limited by the small number of recurrence events, which precluded multivariable predictor modelling, and by the competing risk of death in critically ill patients, which may reduce the opportunity for recurrent VTE to be clinically detected.

Therapeutic anticoagulation was used far more frequently in our ICU cohort than in non-ICU SSPE populations, where fewer than half of patients typically receive full-dose therapy [9–11], with one notable exception. The Dahan et al. study [8] from our institution found that 74% of all hospital SSPE patients received anticoagulation, though notably only 5 of their 166 patients (3%) were in the ICU, and all five ICU patients received anticoagulation. This high overall anticoagulation rate at our institution, even in non-ICU settings, may reflect institutional practice patterns. In the ICU specifically, the near-universal use of anticoagulation likely reflects concern for embolus progression, difficulty assessing symptoms in critically ill patients, and uncertainty about the presence of more proximal thrombus. The competing risks of anticoagulation were clearly apparent: bleeding occurred in 15% of patients, including major bleeding in 12%, rates that exceed those reported in non-ICU SSPE cohorts and are consistent with the high bleeding susceptibility of critically ill patients [18,19]. Bleeding manifestations were heterogeneous and often severe, reflecting the coagulopathy and organ dysfunction common in the ICU. Despite the high use of therapeutic dosing, we found no statistically significant association with any outcome, and no independent predictors of bleeding were identified in multivariable models. Importantly, the markedly different physiologic environment of critical illness, with both enhanced thrombotic drivers and amplified bleeding risk, means that findings from non-ICU cohorts cannot be reliably extrapolated to ICU populations.

### Clinical implications

These findings have several implications for ICU practice. Distal PE appears to be detected more frequently in critically ill patients than in other hospitalized populations [20], likely reflecting a lower threshold for CTPA. VTE recurrence was uncommon, and the small number of events limits any inference regarding predictors of recurrence or the effect of anticoagulation intensity. In contrast, the number of episodes of bleeding underscores the vulnerability of this population to anticoagulant-associated harm. Taken together, these observations highlight the uncertainty surrounding routine use of therapeutic-dose anticoagulation for incidentally identified distal PE in the ICU, particularly in patients without right ventricular dilatation or hemodynamic instability. Given that nearly all patients in our cohort received therapeutic anticoagulation despite the absence of evidence in the literature, current practice appears driven more by perceived risk than by high level evidence. Contemporary guidelines already emphasize individualized assessment for SSPE [6,7], and our findings reinforce the need to consider thrombosis risk, bleeding risk, and overall illness severity when determining anticoagulation strategies in critically ill patients. Ultimately, appropriately designed clinical trials are needed to determine optimal anticoagulation strategies for distal PE in critically ill patients.

### Strengths and limitations

Strengths of this study include being the first ICU-focused evaluation of segmental and SSPE, with comprehensive description of population cohort, extraction of radiologic, clinical, and management data across a 6.5-year period. Multiple analytic approaches, adjusted models, absolute risk differences, and subgroup evaluations, were used to ensure robustness, and missing data were minimal with reliable outcome ascertainment.

This study also has important limitations. Its single-center design and modest sample size limit power. Practice patterns for VTE prophylaxis and CTPA use at a single tertiary center may not reflect a wider practice, thus limiting generalizability. The decision to initiate therapeutic anticoagulation was made by treating physicians. Decisions considered bleeding risk, thrombosis risk, comorbidities, and overall clinical status. Some patients were already receiving prophylactic or therapeutic anticoagulation for other indications prior to CTPA. Thus, treatment allocation was subject to confounding by indication. The small number of bleeding and VTE-recurrence events reduced statistical precision, resulting in wide confidence intervals and non-estimable models. In addition, bleeding risk varied substantially across this heterogeneous ICU population, and we lacked granular data to stratify patients by validated bleeding risk scores. This limits interpretation of safety outcomes. As a retrospective analysis, residual confounding is likely, including variation in imaging thresholds, anticoagulation decisions, and treatment interruptions that were not systematically captured. Formal pre-test probability scores, such as Wells or Geneva scores, were not routinely documented and therefore could not be reliably reconstructed. We instead relied on documented clinical features and radiology request indications, which may incompletely capture the clinical reasoning behind CTPA use. Symptom overlap between PE and critical illness may also have led to detection of clinically insignificant emboli [2], and clot chronicity could not be determined. Due to these unmeasured confounders, the observed lack of association cannot be interpreted as evidence of no effect. Estimates of recurrent VTE were based on crude proportions and did not account for death as a competing event, so recurrence risks may be biased by informative censoring in the context of high in-hospital and 90-day mortality. Recurrent VTE may also have been under detected, as routine follow-up imaging was not performed and symptoms of recurrence may overlap with evolving critical illness. In addition, patients who died early had no opportunity to experience recurrence, potentially biasing recurrence risk estimates downward. These factors should be considered when interpreting the findings.

### Future directions

Prospective studies and pragmatic randomized trials are needed to determine optimal anticoagulation strategies for distal PE in critically ill patients. Future research should consider standardized criteria for CTPA utilization, detailed evaluation of right ventricular function, and structured assessment of bleeding risk. Biomarker- or imaging-based risk models may help identify patients most likely to benefit from therapeutic-dose anticoagulation versus prophylactic dosing. Studies should also account for competing risks, particularly early mortality, and incorporate functional or patient-centered outcomes to better characterize the clinical relevance of distal PE in the ICU. Ultimately, defining which subgroups derive meaningful net benefit from treatment intensification will be essential to balancing thrombotic and haemorrhagic risks in this complex population. Such studies are essential not only to determine whether therapeutic anticoagulation provides benefit, but also to prevent unnecessary harm in patients unlikely to derive advantage from treatment.

## Conclusion

In this cohort of critically ill adults, segmental and SSPE were frequently detected on CTPA and were almost universally managed with therapeutic-dose anticoagulation. Bleeding and VTE recurrence events were infrequent; however, bleeding occurred nearly twice as often as recurrence. There was no statistically significant association between therapeutic-dose anticoagulation and reductions in mortality, bleeding, or recurrence, and right ventricular dilatation was the only factor associated with recurrence risk in exploratory univariable analysis. These findings reveal a disconnect between current practice patterns and the available evidence: despite near-universal use of therapeutic anticoagulation for distal PE in the ICU, we found no clear benefit associated with this approach, while bleeding complications remained substantial. Prospective studies and pragmatic trials are needed to clarify which ICU patients with distal PE truly benefit from therapeutic versus prophylactic anticoagulation. Until such evidence is available, management of distal PE in the ICU will continue to rely largely on clinical judgment rather than robust data.

## Supporting information

S1 File Supplementary appendix, supplementary figures (S1–S4), and supplementary tables (S1–S9). (DOCX)
