# Supplementary material for: Characteristics, management, and outcomes of segmental and subsegmental pulmonary embolism in ICU patients: A retrospective cohort study
Source: PLoS One. 2026 Jul 10;21(7):e0353422. doi: 10.1371/journal.pone.0353422 (PMC13353973; doi:10.1371/journal.pone.0353422)
Supplement: S1 File — (DOCX) [file pone.0353422.s001.docx]

**Supplementary Appendix I**

**The Bleeding Academic Research Consortium (BARC) Definitions**

| Type 0 | No bleeding |
| --- | --- |
| Type 1 | Bleeding that is not actionable and does not cause the patient to seek treatment |
| Type 2 | Any clinically overt sign of hemorrhage that “is actionable” and requires diagnostic studies, hospitalization, or treatment by a health care professional |
| Type 3 | a.       Overt bleeding plus hemoglobin drop of 3 to < 5 g/dL (provided hemoglobin drop is related to bleed); transfusion with overt bleeding  b.      Overt bleeding plus hemoglobin drop ≥ 5 g/dL (provided hemoglobin drop is related to bleed); cardiac tamponade; bleeding requiring surgical intervention for control; bleeding requiring IV vasoactive agents  c.       Intracranial hemorrhage confirmed by autopsy, imaging, or lumbar puncture; intraocular bleed compromising vision |
| Type 4 | CABG-related bleeding within 48 hours |
| Type 5 | a.       Probable fatal bleeding  b.      Definite fatal bleeding (overt or autopsy or imaging confirmation) |

**S1 Fig. Study Flow Diagram**

**
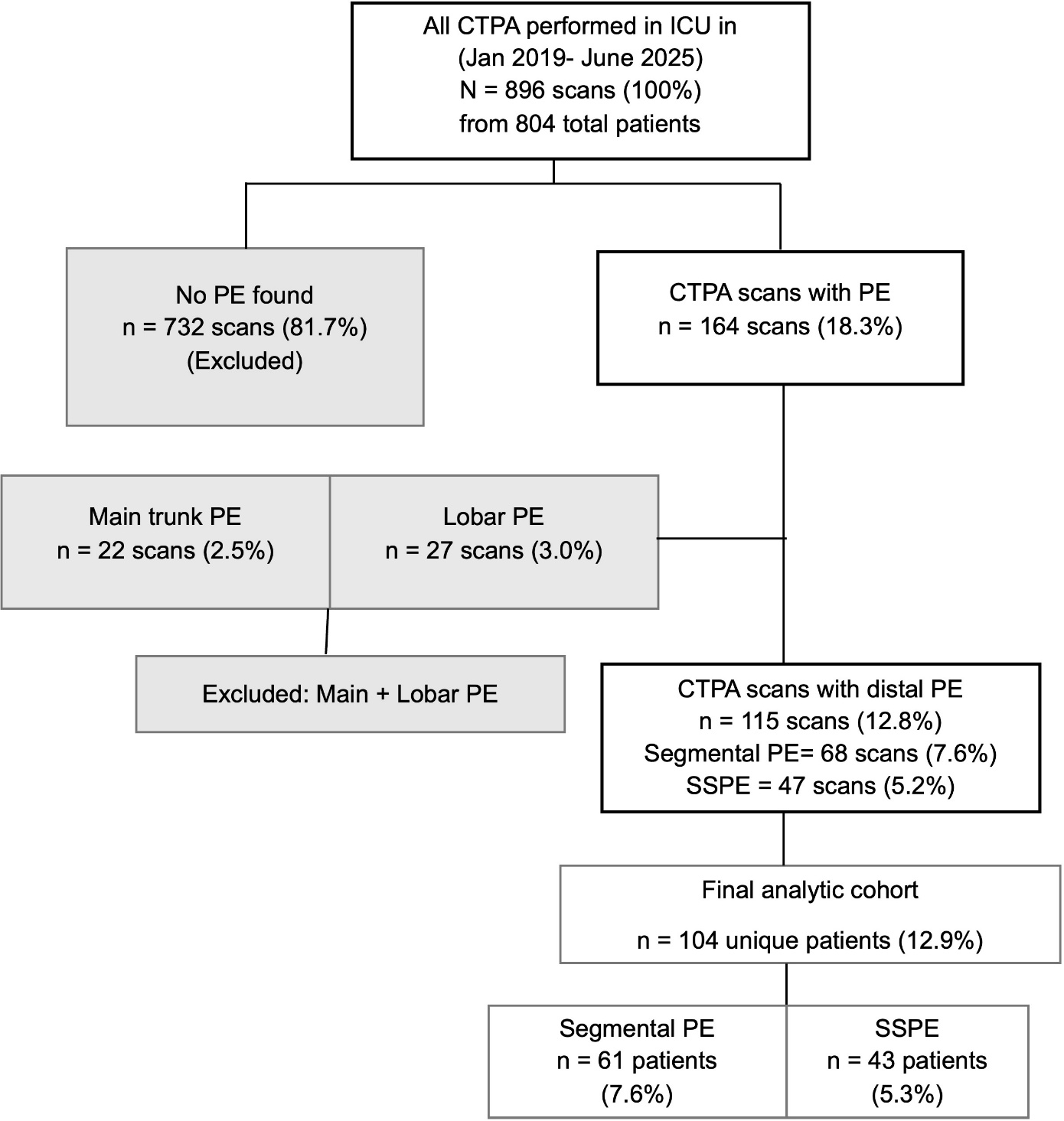
**

A total of 896 computed tomography pulmonary angiography (CTPA) scans were performed in the ICU between January 2019 and June 2025, representing 804 unique patients. Of these, 164 scans (18.3%) demonstrated pulmonary embolism (PE). After excluding main-trunk and lobar PE (49 scans; 5.5%), 115 scans (12.8%) showed distal PE, including 68 scans with segmental PE (7.6%) and 47 scans with subsegmental PE (SSPE) (5.2%). After consolidating multiple scans per individual, the final analytic cohort comprised 104 unique patients with distal PE (61 segmental, 43 SSPE). Abbreviations: PE = pulmonary embolism; SSPE = subsegmental pulmonary embolism; CTPA = computed tomography pulmonary angiography; ICU = intensive care unit.

**S2 Fig. Anticoagulant Type and Continuation After Pulmonary Embolism.**

**
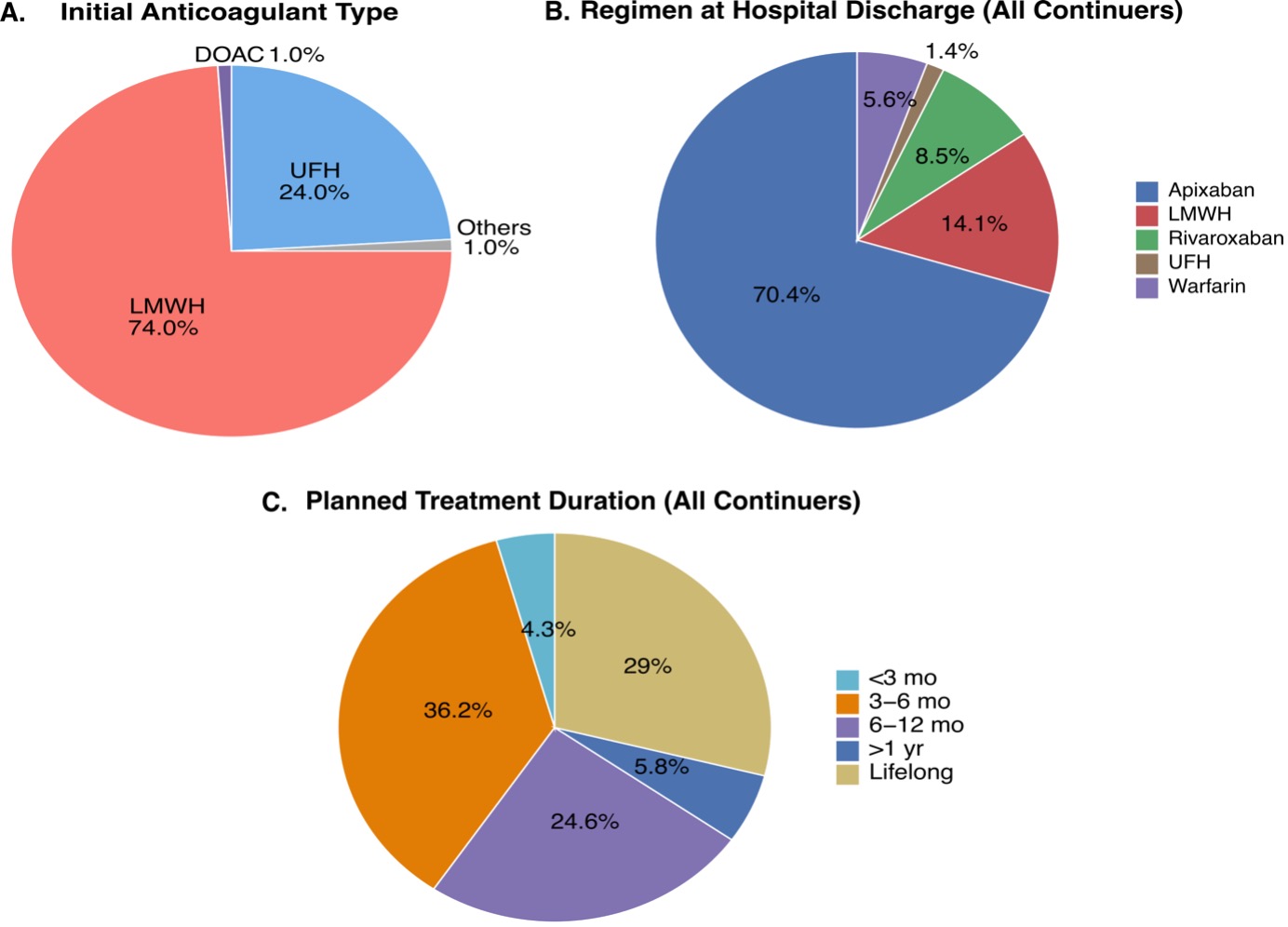
**

Pie charts show the distribution of anticoagulant use among patients with pulmonary embolism. (A) Type of anticoagulant initiated during ICU stay. (B) Anticoagulant class prescribed at hospital discharge. (C) Duration of anticoagulant continuation after discharge. Abbreviation: UFH = unfractionated heparin; LMWH = low-molecular-weight heparin; DOAC = direct oral anticoagulant

**S3 Fig. Absolute Risk Differences Between Therapeutic and Non-Therapeutic Anticoagulation Groups**


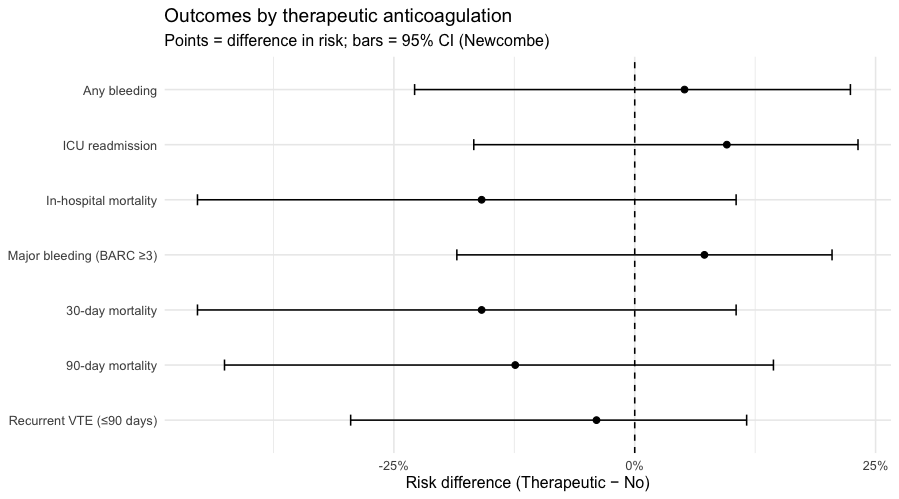


Plot of absolute risk differences (Therapeutic versus No therapeutic) for major clinical outcomes. Points represent the difference in event risk; bars indicate 95% confidence intervals calculated using the Newcombe method. Negative values indicate lower risk in the therapeutic anticoagulation group. *Abbreviations:* BARC = Bleeding Academic Research Consortium; ICU = intensive care unit; VTE = venous thromboembolism.

### ****S4 Fig. Risk differences by therapeutic anticoagulation in patients with subsegmental and segmental pulmonary embolism.****


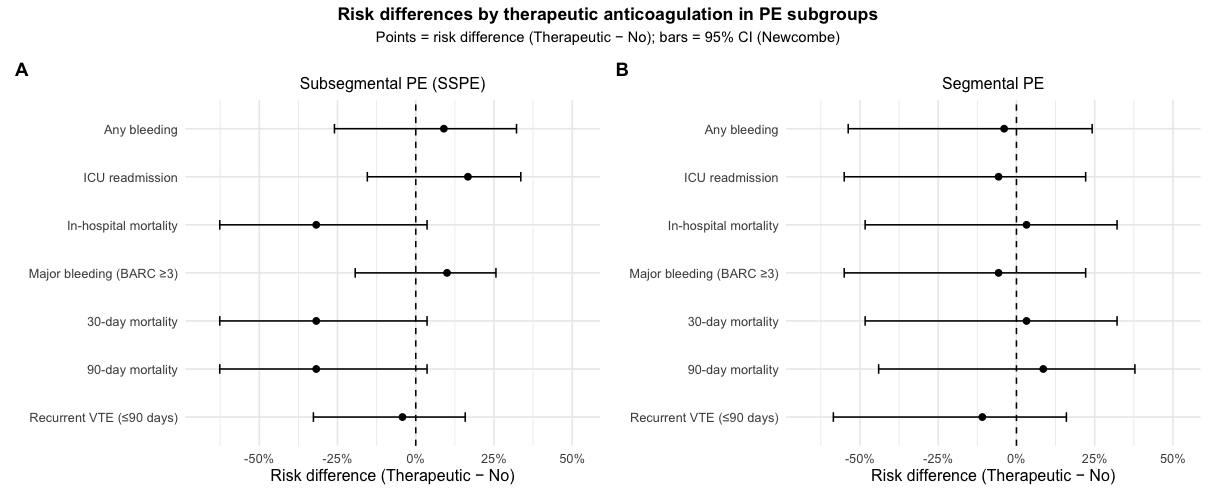


Forest plots display absolute risk differences (therapeutic − no therapeutic) with 95% confidence intervals (CIs) for key clinical outcomes, including any bleeding, major bleeding (BARC ≥ 3), ICU readmission, in-hospital mortality, 30-day mortality, 90-day mortality, and recurrent venous thromboembolism (VTE) within 90 days for (A) subsegmental pulmonary embolism (SSPE) and (B) segmental pulmonary embolism. Risk difference was calculated as therapeutic − no therapeutic (positive values indicate higher risk in the therapeutic group). Confidence intervals were computed using the Newcombe hybrid score method with Wilson intervals for each group. *Abbreviations:* ICU, Intensive Care Unit; PE, pulmonary embolism; SSPE, subsegmental pulmonary embolism; VTE, venous thromboembolism; BARC, Bleeding Academic Research Consortium; CI, confidence interval.

**S1 Table. Characteristics of Patients with Documented Bleeding After Anticoagulation**

| **No** | **PE type** | **Anticoagulation Dose** | **Bleeding site** | **Onset from anticoagulation (days)** | **BARC grade** | **Hemoglobin drop (g/dL)** | **Transfusion required** | **Intervention required** | **RBC units transfused** |
| --- | --- | --- | --- | --- | --- | --- | --- | --- | --- |
| 1 | Segmental | Therapeutic | Intrathoracic | 34.6 | 5 | 12 → 6.8 | Yes | Yes | 21 |
| 2 | Segmental | Therapeutic | Intra-abdominal | 59.0 | 3b | 8.0 → 6.8 | Yes | Yes | 4 |
| 3 | Segmental | Therapeutic | Hemoptysis | 4.3 | 3a | 11.7 → 8.5 | No | No | – |
| 4 | Subsegmental | Therapeutic | Retroperitoneal | 3.2 | 3b | 10.8 → 8.3 | Yes | Yes | 2 |
| 5 | Subsegmental | Therapeutic | Retroperitoneal | 21.3 | 3b | 8.1 → 6.4 | Yes | Yes | 5 |
| 6 | Segmental | Therapeutic | External | 7.1 | 2 | – | No | No | – |
| 7 | Subsegmental | Therapeutic | Intra-abdominal | 1.3 | 3a | 9.3 → 8.3 | Yes | No | 1 |
| 8 | Segmental | Therapeutic | Gastrointestinal | 1.5 | 3b | 9.1 → 7.1 | Yes | Yes | 1 |
| 9 | Segmental | Therapeutic | Hemoptysis | 26.2 | 3b | 9.6 → 7.8 | Yes | Yes | 4 |
| 10 | Segmental | Therapeutic | Intramuscular | 2.5 | 3a | 12.2 → 8.8 | Yes | No | 3 |
| 11 | Subsegmental | Therapeutic | Hemoptysis | 1.2 | 2 | 10.1 → 8.8 | No | No | – |
| 12 | Subsegmental | Therapeutic | External | 0 | 2 | – | No | No | – |
| 13 | Subsegmental | Prophylactic | Hemoptysis | 31.1 | 2 | – | No | No | – |
| 14 | Segmental | Therapeutic | Intra-abdominal | 19.3 | 3a | 10.7 → 5.9 | Yes | No | 6 |
| 15 | Segmental | Therapeutic | Intracerebral | 4.6 | 3c | – | Yes | Yes | 1 |
| 16 | Segmental | Prophylactic | Intramuscular | 59.4 | 3a | 9.0 → 7.0 | Yes | No | 5 |

Data are presented for all patients with documented bleeding events following anticoagulation. *Abbreviations:* BARC = Bleeding Academic Research Consortium; CTPA = computed tomography pulmonary angiography; PE = pulmonary embolism; RBC = red blood cell; SSPE = subsegmental pulmonary embolism. “Onset from anticoagulation” denotes the interval from the first anticoagulant dose to the time of bleeding diagnosis.

**S2 Table. Characteristics of Patients with Recurrent Venous Thromboembolism (VTE) After CTPA**

| **No** | **PE type** | **Onset (days after CTPA)** | **VTE recurrence** | | **Recurrent VTE type** | **Anticoagulation Dose** | **Duration of anticoagulation after discharge** | **DVT on Doppler** |
| --- | --- | --- | --- | --- | --- | --- | --- | --- |
|  |  |  | **during same admission** | **Within 90 days** |  |  |  |  |
| 1 | Segmental | 11 | Yes | Deceased | IVC thrombus / massive PE | Therapeutic | Deceased | Not done |
| 2 | Subsegmental | 85 | No | Yes | DVT (right proximal leg) | Prophylactic | 1 year | Not done |
| 3 | Segmental | 9 | Yes | Deceased | New SSPE | Therapeutic | Deceased | No |
| 4 | Segmental | 18 | No | Yes | New SSPE | Therapeutic | 6 months | No |
| 5 | Subsegmental | 7 | Yes | No | New lobar PE | Therapeutic | 1 year | Yes |
| 6 | Segmental | 28 | Yes | No | New segmental PE | Therapeutic | Lifelong | No |
| 7 | Segmental | 49 | Yes | Yes | New segmental PE | Prophylactic | 3 months | Yes |
| 8 | Segmental | 5 | Yes | Yes | New segmental PE | Therapeutic | 3 months | No |

Data are presented for patients with recurrent venous thromboembolism (VTE) identified after the initial computed tomography pulmonary angiography (CTPA). “Onset of recurrent VTE (days after initial CTPA)” indicates the interval between the date of the first CTPA confirming pulmonary embolism and the diagnosis of a subsequent VTE event, either during the same hospital admission or after discharge. *Abbreviations:* CTPA = computed tomography pulmonary angiography; DVT = deep-vein thrombosis; PE = pulmonary embolism; SSPE = subsegmental pulmonary embolism.

**S3 Table. Adjusted odds ratios for major clinical outcomes associated with therapeutic anticoagulation in the full cohort and by pulmonary embolism (PE) subgroup.**

| **Outcome** | **Events/N (analysis set)** | **Full cohort  (aOR 95% CI)** | ***P* value** |  | ***P* value** | **SSPE  (aOR 95% CI)** | ***P* value** |
| --- | --- | --- | --- | --- | --- | --- | --- |
| Any bleeding | 16/104 | 2.34 (0.47–19.2) | 0.35 | 1.96 (0.13–76.1) | 0.66 | NE | - |
| Major bleeding (BARC ≥3) | 12/104 | 6.45 (0.72–172) | 0.16 | 1.66 (0.08–83.8) | 0.77 | NE | - |
| Recurrent VTE ≤90 days | 8/102 | 0.69 (0.11–6.22) | 0.71 | 0.71 (0.04–28.4) | 0.83 | 0.24  (0.005–9.88) | 0.42 |
| 90-day mortality | 24/98 | 0.70 (0.21–2.45) | 0.56 | 2.93 (0.32–69.2) | 0.40 | NE | - |

Models adjusted for age, active cancer, chronic kidney disease (CKD), right-ventricular (RV) dilatation, and concomitant deep-vein thrombosis (DVT). Outcome ascertainment at 90 days: mortality—24/104 died, 74/104 alive, 6/104 unknown; recurrence—8/102 recurred, 72/102 no recurrence, 2/102 no record; 20/102 died before day 90 (treated as a competing event). Denominators vary across outcomes due to listwise deletion from missing covariates in multivariable models. “NE” denotes non-estimable effects owing to quasi-separation or model non-convergence. Abbreviations: aOR, adjusted odds ratio; BARC, Bleeding Academic Research Consortium; CI, confidence interval; CKD, chronic kidney disease; DVT, deep-vein thrombosis; PE, pulmonary embolism; RV, right ventricle; SSPE, subsegmental pulmonary embolism; VTE, venous thromboembolism.

**S4 Table. Absolute risks and risk differences for major outcomes by therapeutic anticoagulation**

| **Outcome** | **Therapeutic AC** | **No therapeutic AC** | **Risk difference (95% CI)** |
| --- | --- | --- | --- |
| Any bleeding | 14/86 (16.3%) | 2/18 (11.1%) | 5.2% (−22.8%, 22.4%) |
| ICU readmission | 13/86 (15.1%) | 1/18 (5.6%) | 9.6% (−16.7%, 23.2%) |
| In-hospital mortality | 15/86 (17.4%) | 6/18 (33.3%) | −15.9% (−45.4%, 10.5%) |
| Major bleeding (BARC ≥3) | 11/86 (12.8%) | 1/18 (5.6%) | 7.2% (−18.5%, 20.5%) |
| 30-day mortality | 15/86 (17.4%) | 6/18 (33.3%) | −15.9% (−45.4%, 10.5%) |
| 90-day mortality | 18/86 (20.9%) | 6/18 (33.3%) | −12.4% (−42.6%, 14.4%) |
| Recurrent VTE (≤90 days)† | 6/84 (7.1%) | 2/18 (11.1%) | −4.0% (−29.5%, 11.6%) |

Risk difference is calculated as **Therapeutic − No therapeutic** (positive values favor the No-therapy group; negative values favor the Therapeutic group). Confidence intervals are Newcombe hybrid scores using Wilson intervals for each group.
†Denominator differs because two patients in the therapeutic AC group had no available record for 90-day recurrent VTE (n = 84). *Abbreviations:* AC = anticoagulation; VTE = venous thromboembolism; BARC = Bleeding Academic Research Consortium; CI = confidence interval.

**S5 Table. Absolute risks and risk differences for major outcomes by therapeutic anticoagulation in patients with segmental and subsegmental pulmonary embolism (PE)**

| **Outcome** | **Therapeutic AC** | **No therapeutic AC** | **Risk difference (95% CI)** |
| --- | --- | --- | --- |
| **Segmental PE (n = 61)** | | | |
| Any bleeding | 9/56 (16.1%) | 1/5 (20.0%) | −3.9% (−53.8%, 24.2%) |
| ICU readmission | 8/56 (14.3%) | 1/5 (20.0%) | −5.7% (−55.0%, 22.1%) |
| In-hospital mortality | 13/56 (23.2%) | 1/5 (20.0%) | 3.2% (−48.3%, 32.1%) |
| Major bleeding (BARC ≥3) | 8/56 (14.3%) | 1/5 (20.0%) | −5.7% (−55.0%, 22.1%) |
| 30-day mortality | 13/56 (23.2%) | 1/5 (20.0%) | 3.2% (−48.3%, 32.1%) |
| 90-day mortality | 16/56 (28.6%) | 1/5 (20.0%) | 8.6% (−44.0%, 37.9%) |
| Recurrent VTE (≤90 days) | 5/55 (9.1%) | 1/5 (20.0%) | −10.9% (−58.5%, 16.0%) |
| **Subsegmental PE (n = 43)** | | | |
| Any bleeding | 5/30 (16.7%) | 1/13 (7.7%) | 9.0% (−26.0%, 32.2%) |
| ICU readmission | 5/30 (16.7%) | 0/13 (0.0%) | 16.7% (−15.5%, 33.6%) |
| In-hospital mortality | 2/30 (6.7%) | 5/13 (38.5%) | −31.8% (−62.6%, 3.6%) |
| Major bleeding (BARC ≥3) | 3/30 (10.0%) | 0/13 (0.0%) | 10.0% (−19.4%, 25.6%) |
| 30-day mortality | 2/30 (6.7%) | 5/13 (38.5%) | −31.8% (−62.6%, 3.6%) |
| 90-day mortality | 2/30 (6.7%) | 5/13 (38.5%) | −31.8% (−62.6%, 3.6%) |
| Recurrent VTE (≤90 days)† | 1/29 (3.4%) | 1/13 (7.7%) | −4.2% (−32.7%, 15.8%) |

Risk difference was calculated as **Therapeutic − No therapeutic** (positive values indicate higher risk in the therapeutic group). Confidence intervals were derived using the Newcombe method with Wilson score intervals for each group.
†Denominator differs because one patient in the therapeutic AC group had no available record for 90-day recurrent VTE (n = 29). *Abbreviations:* AC = anticoagulation; PE = pulmonary embolism; VTE = venous thromboembolism; BARC = Bleeding Academic Research Consortium; CI = confidence interval; ICU, Intensive Care Unit.

**S6 Table. Univariable logistic regression for 90-day recurrent venous thromboembolism (n = 8/102)**

| **Predictor** | **Level / Comparison** | **OR (95% CI)** | ***p*-value** |
| --- | --- | --- | --- |
| Age | per year increase | 0.98 (0.94 – 1.03) | 0.50 |
| Sex | Male vs Female | 0.54 (0.12 – 2.42) | 0.41 |
| Active cancer | Yes vs No | 2.08 (0.28 – 15.1) | 0.40 |
| PE lobe | Multiple vs Single | 0.89 (0.17 – 3.86) | 0.88 |
| Concomitant DVT | Yes vs No | 1.51 (0.21 – 7.23) | 0.63 |
| COVID-19 infection | Yes vs No | 1.62 (0.22 – 7.82) | 0.57 |
| RV dilation on CTPA | Yes vs No | 10.3 (1.50 – 88.3) | 0.018 |
| PE location | Subsegmental vs Segmental | 0.45 (0.06 – 2.07) | 0.34 |
| Therapeutic anticoagulation in ICU | Yes vs No | 0.62 (0.13 – 4.55) | 0.57 |
| Continued anticoagulation after discharge | Yes vs No | 1.25 (0.27 – 9.04) | 0.79 |

Abbreviations: OR = odds ratio; CI = confidence interval; PE = Pulmonary Embolism; DVT = deep-vein thrombosis; RV = right ventricular; CTPA = computed tomography pulmonary angiography; SSPE = subsegmental pulmonary embolism; ICU, Intensive Care Unit. Note: Eight recurrence events (7.8 %) were observed. Models are univariable; estimates are exploratory owing to sparse events and should be interpreted with caution.

**S7 Table. Univariable logistic regression for any in-ICU bleeding (n = 16 / 104, 15.4 %)**

| **Predictor** | **Level / Comparison** | **OR (95 % CI)** | **p-value** |
| --- | --- | --- | --- |
| Age | per year increase | 0.99 (0.96 – 1.03) | 0.64 |
| Sex | Male vs Female | 0.95 (0.32 – 3.02) | 0.93 |
| Active cancer | Yes vs No | 1.46 (0.30 – 5.41) | 0.59 |
| Chronic kidney disease | Yes vs No | 1.89 (0.09 – 15.9) | 0.59 |
| COVID-19 infection | Yes vs No | 0.28 (0.01 – 1.53) | 0.23 |
| Concomitant DVT | Yes vs No | 1.39 (0.36 – 4.58) | 0.60 |
| PE location | Subsegmental vs Segmental | 0.83 (0.26 – 2.43) | 0.73 |
| Therapeutic anticoagulation during ICU | Yes vs No | 1.56 (0.38 – 10.5) | 0.58 |
| Anticoagulant type (LMWH vs Other) | LMWH vs Other | 0.27 (0.09 – 0.83) | 0.022 |
| Over-therapeutic level | Yes vs No | 1.43 (0.35 – 5.16) | 0.60 |
| Continued anticoagulation after discharge | Yes vs No | 1.25 (0.39 – 4.77) | 0.72 |
| RV dilation on CTPA | Yes vs No | 6.07 (1.43 – 26.1) | 0.013 |

Abbreviations: OR = odds ratio; CI = confidence interval; DVT = deep-vein thrombosis; RV = right ventricular; CTPA = computed tomography pulmonary angiography; LMWH = low-molecular-weight heparin; ICU, Intensive Care Unit. Note: Sixteen bleeding events (15 %) occurred. All analyses are univariable and exploratory. Odds ratios represent the comparison shown (e.g., Yes vs No). Estimates with wide confidence intervals reflect sparse data and should be interpreted cautiously.

**S8 Table. Firth penalized logistic regression for predictors of any in-ICU bleeding
(n = 100)**

| **Predictor** | **Level / Comparison** | **Adjusted OR (95 % CI)** | **p-value** |
| --- | --- | --- | --- |
| RV dilation on CTPA | Yes vs No | 3.94 (0.91 – 16.7) | 0.066 |
| Anticoagulant type (LMWH vs Other) | LMWH vs Other | 0.35 (0.11 – 1.11) | 0.075 |

**Model summary:** Likelihood ratio test = 9.49 on 3 df, *p* = 0.023; *n* = 100.
Abbreviations: OR = odds ratio; CI = confidence interval; CTPA = computed tomography pulmonary angiography; LMWH = low-molecular-weight heparin. Note: Variables with *p* < 0.10 in univariable analysis were included in the model. Sixteen bleeding events (15 %) occurred among 104 patients; four patients not receiving anticoagulation were excluded because the variable “anticoagulant type” was not applicable. Firth penalized logistic regression was used to reduce small-sample bias; results are exploratory.

**S9 Table**. **Sensitivity Analyses of Clinical Outcomes and Resource Use According to Therapeutic Anticoagulation Dose**

| **Outcome** | **Whole cohort** | | **p** | **SSPE** | | **p** | **Segmental PE** | | **p** |
| --- | --- | --- | --- | --- | --- | --- | --- | --- | --- |
|  | **Therapeutic AC (n=86)** | **Non-therapeutic AC (n=18)** |  | **Therapeutic AC (n=30)** | **Non-therapeutic AC (n=13)** |  | **Therapeutic AC (n=56)** | **Non-therapeutic AC (n=5)** |  |
| Any bleeding after CTPA | 14 (16%) | 2 (11%) | 0.73 | 5 (17%) | 1 (7.7%) | 0.65 | 9 (16%) | 1 (20%) | >0.99 |
| Major bleeding (BARC ≥3) | 11 (13%) | 1 (5.6%) | 0.69 | 3 (10%) | 0 (0%) | 0.54 | 8 (14%) | 1 (20%) | 0.56 |
| RBC transfusion required for the bleeding | 10 (67%) | 1 (50%) | >0.99 | 3 (60%) | 0 (0%) | >0.99 | 7 (70%) | 1 (100%) | >0.99 |
| Intervention required to stop bleeding | 7 (50%) | 0 (0%) | 0.48 | 2 (40%) | 0 (0%) | >0.99 | 5 (56%) | 0 (0%) | >0.99 |
| Recurrent VTE during same hospital admission | 5 (5.8%) | 1 (5.6%) | >0.99 | 1 (3.3%) | 0 (0%) | >0.99 | 4 (7.1%) | 1 (20%) | 0.36 |
| Recurrent VTE within 90 days | 6 (7.1%) | 2 (11%) | 0.63 | 1 (3.4%) | 1 (7.7%) | 0.53 | 5 (9.1%) | 1 (20%) | 0.42 |
| In-hospital mortality | 15 (17%) | 6 (33%) | 0.19 | 2 (6.7%) | 5 (38%) | 0.02 | 13 (23%) | 1 (20%) | >0.99 |
| 90-day mortality | 18 (22%) | 6 (35%) | 0.35 | 2 (6.9%) | 5 (42%) | 0.02 | 16 (31%) | 1 (20%) | >0.99 |
| ICU length of stay, days | 7.1 [3.1–16.3] | 8.6 [3.9–14.9] | 0.51 | 5.8 [2.8–10.4] | 7.1 [3.8–13.2] | 0.77 | 8.2 [3.1–20.4] | 14.9 [13.0–30.6] | 0.09 |
| Hospital length of stay, days | 21.6 [12.2–39.4] | 23.0 [15.8–44.4] | 0.93 | 15.0 [11.0–47.4] | 22.0 [7.4–27.7] | 0.99 | 26.0 [13.5–37.7] | 44.4 [15.9–47.8] | 0.35 |

Data are presented as median [IQR] or n (%) unless otherwise indicated. P values were calculated using Fisher’s exact test for categorical variables and the Wilcoxon rank-sum test for continuous variables. Abbreviations: BARC = Bleeding Academic Research Consortium; CTPA = computed tomography pulmonary angiography; ICU = intensive care unit; IQR = interquartile range; PE = pulmonary embolism; RBC = red blood cell; SSPE = subsegmental pulmonary embolism; VTE = venous thromboembolism
